# Live enteroviruses, but not other viruses, detected in human pancreas at the onset of type 1 diabetes in the DiViD study

**DOI:** 10.1007/s00125-022-05779-2

**Published:** 2022-08-12

**Authors:** Lars Krogvold, Angelo Genoni, Anna Puggioni, Daniela Campani, Sarah J. Richardson, Christine S. Flaxman, Bjørn Edwin, Trond Buanes, Knut Dahl-Jørgensen, Antonio Toniolo

**Affiliations:** 1 Division of Pediatric and Adolescent Medicine, Oslo University Hospital, Oslo, Norway; 2 Institute of Clinical Dentistry, Faculty of Dentistry, University of Oslo, Oslo, Norway; 3 Department of Biotechnology and Life Sciences, University of Insubria, Varese, Italy; 4 Department of Surgical, Medical and Molecular Pathology and Critical Care, University of Pisa, Pisa, Italy; 5 Islet Biology Group (IBEx), Exeter Centre of Excellence in Diabetes (EXCEED), University of Exeter College of Medicine and Health, Exeter, UK; 6 Department for HPB Surgery, Oslo University Hospital, Oslo, Norway; 7 Institute of Clinical Medicine, Faculty of Medicine, University of Oslo, Oslo, Norway; 8 Global Virus Network, University of Insubria, Varese, Italy

**Keywords:** Biopsy, Cell culture, Enterovirus, Gene amplification, Immunofluorescence, Pancreas, Type 1 diabetes

## Abstract

**Aims/hypothesis:**

Enterovirus (EV) infection of pancreatic islet cells is one possible factor contributing to type 1 diabetes development. We have reported the presence of EV genome by PCR and of EV proteins by immunohistochemistry in pancreatic sections. Here we explore multiple human virus species in the Diabetes Virus Detection (DiViD) study cases using innovative methods, including virus passage in cell cultures.

**Methods:**

Six recent-onset type 1 diabetes patients (age 24–35) were included in the DiViD study. Minimal pancreatic tail resection was performed under sterile conditions. Eleven live cases (age 43–83) of pancreatic carcinoma without diabetes served as control cases. In the present study, we used EV detection methods that combine virus growth in cell culture, gene amplification and detection of virus-coded proteins by immunofluorescence. Pancreas homogenates in cell culture medium were incubated with EV-susceptible cell lines for 3 days. Two to three blind passages were performed. DNA and RNA were extracted from both pancreas tissue and cell cultures. Real-time PCR was used for detecting 20 different viral agents other than EVs (six herpesviruses, human polyomavirus [BK virus and JC virus], parvovirus B19, hepatitis B virus, hepatitis C virus, hepatitis A virus, mumps, rubella, influenza A/B, parainfluenza 1–4, respiratory syncytial virus, astrovirus, norovirus, rotavirus). EV genomes were detected by endpoint PCR using five primer pairs targeting the partially conserved 5′ untranslated region genome region of the A, B, C and D species. Amplicons were sequenced. The expression of EV capsid proteins was evaluated in cultured cells using a panel of EV antibodies.

**Results:**

Samples from six of six individuals with type 1 diabetes (cases) and two of 11 individuals without diabetes (control cases) contained EV genomes (*p*<0.05). In contrast, genomes of 20 human viruses other than EVs could be detected only once in an individual with diabetes (Epstein–Barr virus) and once in an individual without diabetes (parvovirus B19). EV detection was confirmed by immunofluorescence of cultured cells incubated with pancreatic extracts: viral antigens were expressed in the cytoplasm of approximately 1% of cells. Notably, infection could be transmitted from EV-positive cell cultures to uninfected cell cultures using supernatants filtered through 100 nm membranes, indicating that infectious agents of less than 100 nm were present in pancreases. Due to the slow progression of infection in EV-carrying cell cultures, cytopathic effects were not observed by standard microscopy but were recognised by measuring cell viability. Sequences of 5′ untranslated region amplicons were compatible with EVs of the B, A and C species. Compared with control cell cultures exposed to EV-negative pancreatic extracts, EV-carrying cell cultures produced significantly higher levels of IL-6, IL-8 and monocyte chemoattractant protein-1 (MCP1).

**Conclusions/interpretation:**

Sensitive assays confirm that the pancreases of all DiViD cases contain EVs but no other viruses. Analogous EV strains have been found in pancreases of two of 11 individuals without diabetes. The detected EV strains can be passaged in series from one cell culture to another in the form of poorly replicating live viruses encoding antigenic proteins recognised by multiple EV-specific antibodies. Thus, the early phase of type 1 diabetes is associated with a low-grade infection by EVs, but not by other viral agents.

**Graphical abstract:**

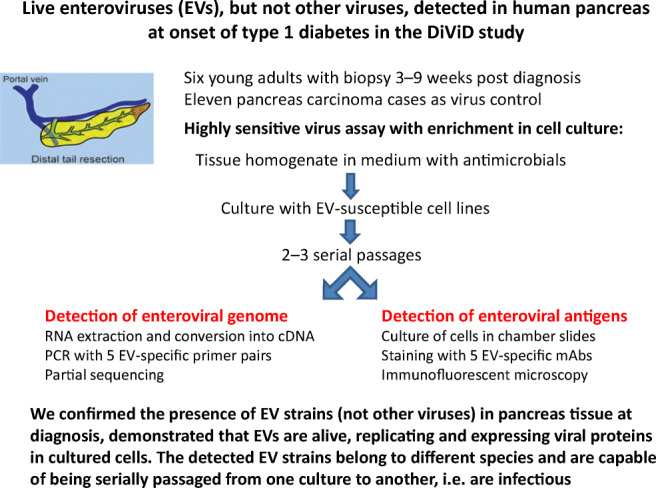

**Supplementary Information:**

The online version contains peer-reviewed but unedited supplementary material available at 10.1007/s00125-022-05779-2.

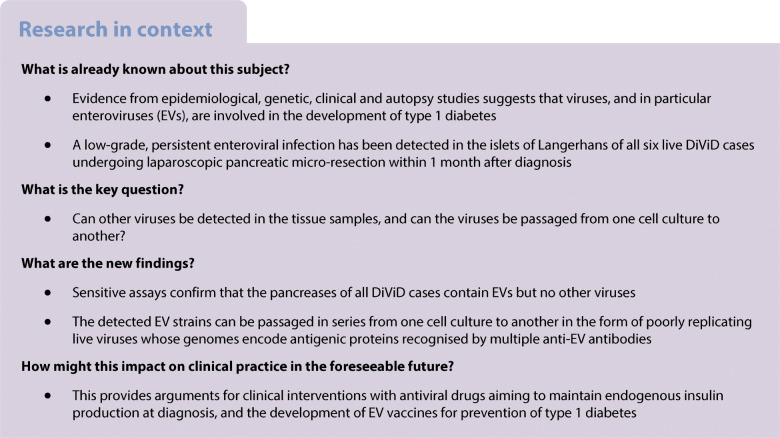



## Introduction

Type 1 diabetes is characterised by the loss of insulin-producing pancreatic beta cells due to a complex interaction of genetic, immunologic and environmental factors. Numerous studies have associated type 1 diabetes with an enteroviral infection which could act through cytolytic effects on beta cells and through bystander activation of immune factors [1]. Enteroviruses (EVs) are members of the family Picornaviridae, genus *Enterovirus*: non-enveloped virus particles of about 30 nm, with a capsid surrounding the positive-sense single-stranded RNA genome. About 240 EV types capable of infecting humans are reported (https://www.picornaviridae.com/).

Notably, EV capsid antigens have been detected post-mortem in pancreatic islets of type 1 diabetes cases [2, 3] and in pancreatic biopsies from live participants in the Diabetes Virus Detection (DiViD) study [4]. At the time of clinical onset, IFN-stimulated genes (ISGs) are expressed in the islets [5], HLA class I is hyperexpressed [4, 6] and memory CD8 T lymphocytes are among the infiltrating cells [7]. Serologic studies showed that neutralising antibodies to group B coxsackieviruses (EV genus) are associated with the initiation of beta cell autoimmunity that heralds diabetes [8]. In addition, studies of children genetically predisposed to type 1 diabetes have shown that EVs of different types are present in stools before the development of islet autoantibodies [9, 10]. The data indicate that EV infection represents an important environmental factor for type 1 diabetes. Results of collaborative studies reinforce this notion, showing that EV infection can persist in the pancreas and lymphoid tissue of type 1 diabetes patients for a long time after diagnosis (unpublished data—nPOD-V consortium, https://www.jdrfnpod.org/publications/npod-working-groups/npod-viral-work-group). Thus, type 1 diabetes patients seem affected by a persistent, low-grade EV infection [4, 11, 12].

Acute EV infections rapidly shut off protein synthesis, producing cell death [13], whereas persistent EV infections are attributed to virus variants endowed with little replicative ability [11, 12] and capable of egressing the cell without lysis [13]. These EV variants are characterised by continuous release of particles without manifest cytopathology and enduring activation of immune-related genes [14].

In addition to EVs, other viruses have been associated with type 1 diabetes [15, 16], especially rotavirus infection [17] and congenital rubella [18]. Small studies and case reports have also associated type 1 diabetes or fulminant diabetes with coxsackieviruses A and B (coxsackievirus A2 [CAV-2] [19], coxsackievirus B [CBV]1 [20], CBV4 [21]), influenza virus [22], parainfluenza virus type 3 [19], mumps virus [23], cytomegalovirus (CMV) [24], Epstein–Barr virus (EBV) [25] and human herpesvirus (HHV)-6 [26]. Alterations of glucose homeostasis have been reported also in hepatitis C, where metabolic changes may be reversed by antivirals [27].

Due to the lack of available pancreas samples obtained at the time of the clinical onset, there is limited information on which viruses might harm the islets in type 1 diabetes. The DiViD study was initiated to obtain pancreatic biopsies from adults recently diagnosed with type 1 diabetes [28].

The DiViD reports published so far have shown that: (1) the six DiViD participants are EV-positive by RT-PCR in at least one of the following samples: purified islets, peripheral blood mononuclear cells (PBMCs), duodenal biopsy, stool [4, 29]; (2) insulitis and a reserve of insulin-producing cells are still present at the time of diagnosis [30]; (3) in vitro, the potential of isolated islets to produce insulin remains adequate [31]; (4) in insulitic lesions, a substantial proportion of infiltrating CD8^+^ T cells bear the phenotype of tissue-resident memory T cells, not classic cytotoxic CD8^+^ T cells [7], with very few CD20^+^ B cells (CD20Lo immunophenotype) [32]; (5) ISGs are overexpressed in islets [5]; and (6) the deposition of amyloid in islets is already present in the initial phase of diabetes [33].

Yet, the above studies did not investigate the possible involvement of viral agents other than EVs. The present study was designed to explore the association of diabetes with EV infections and to evaluate if other human viruses were contributing to the disease. To this end, pancreas samples of the DiViD study have been re-analysed for EVs and additional viral agents: herpesviruses, parvovirus B19, polyomaviruses, hepatitis viruses, respiratory viruses and gastrointestinal viruses.

## Methods

Consumables, chemicals, cell lines, viruses, culture media, antibodies, reagents for molecular biology, commercial PCR kits, instruments, virus databases, software are listed in electronic supplementary material (ESM) Table 1.

### Clinical cases

As shown in ESM Table 2, six type 1 diabetes patients (three women, three men; 24–35 years of age) were recruited in the DiViD study (Oslo, Norway) between 3 and 9 weeks after diagnosis (median 5 weeks). The DiViD study was approved by the Norwegian Government’s Regional Ethics Committee and written informed consent was obtained from participants. Pancreatic biopsies, consisting of a 2 to 3-cm-long piece of the tail of each pancreas, were taken by laparoscopy under general anaesthesia. Biopsies were dissected in sterile conditions and snap-frozen in liquid nitrogen in the operation theatre. Formalin-fixed paraffin-embedded (FFPE) tissue blocks were also prepared. All participants were insulin-dependent at the time of biopsy and were treated with 0.16–0.53 (mean 0.37) U kg^−1^ day^−1^. As published, all were positive for GAD autoantibodies, and five were positive for three out of four autoantibodies [28]. All had at least one high-risk HLA haplotype [28].

Eleven individuals without diabetes from the Pancreatic Carcinoma Study at the University of Pisa (Pisa, Italy) contributed frozen and FFPE tissue obtained at surgery. Participant age was 43–83 years (median 68 years). Diabetes-related autoantibodies and HLA typing were not available (ESM Table 2).

### Pancreas tissue samples

The virology study of pancreatic samples was approved by the Ethics Committee of Ospedale di Circolo and Fondazione Macchi (Varese, Italy; no. 17233/2018-10-30) and performed in accordance with the Declaration of Helsinki and local regulatory laws. For virus isolation, pancreas samples from the DiViD study (Oslo, Norway) and the Pancreatic Carcinoma Study (Pisa, Italy) were shipped in liquid nitrogen to the laboratory. Aliquots of each sample (approximately 100 mg) were used for virus detection. Tissue was homogenised in cell culture medium supplemented with a mixture of antibacterial/antifungal drugs (PANTA) using glass beads (MP Fast Prep-24). Residual tissue samples were frozen at −70°C for subsequent analysis.

### Immunohistochemistry

Immunohistochemistry (IHC) was performed using a previously reported immunoperoxidase staining method for islet hormones and the EV capsid protein VP1 (monoclonal antibody [mAb] 5D8/1) [4].

### Cell lines and virus strains

The human cell lines AV3, RD, VC3 and HEK-293 were obtained from European Collection of Authenticated Cell Cultures (ECACC); the monkey cell line LLC-MK2 was from American Type Culture Collection (ATCC). Cells were cultured in DMEM/F12 medium supplemented with l-glutamine, heat-inactivated 10% fetal bovine serum and penicillin/streptomycin. Cell cultures were monitored for mycoplasma contamination (MycoAlert Plus Mycoplasma kit). The Nancy strain of CBV3 obtained from ATCC was propagated in AV3 cells, titrated and stored at −70°C. CBV3 was used as a virus control for PCR and immunofluorescence (IF) assays.

### Detection of multiple viral agents in pancreas samples

For each case, nucleic acids were extracted using four 0.6 ml aliquots of pancreas homogenate. An automated m2000sp system based on magnetic nanoparticles was employed with reagent kits specific for RNA or DNA (Abbott Molecular, Rome, Italy). RNA was reverse transcribed as reported below. DNA was used directly. For each sample, 15 μl of template DNA was tested in duplicate. Real-time quantitative PCR kits (analytical sensitivity 10–100 genome equivalents per reaction) were used (ESM Table 1): hepatitis B virus (HBV), hepatitis C virus (HCV), CMV, EBV, BK polyomavirus; varicella-zoster virus (VZV), HHV-6, JC polyomavirus, parvovirus B19, influenza virus A and B, parainfluenza virus 1–4, respiratory syncytial virus (RSV) A and B, astrovirus species 1–8, norovirus genogroups I and II, rotavirus, hepatitis A virus (HAV), EVs; HHV-7; rubella virus; and mumps virus. Real-time PCR reactions were run on ABI Prism 7500 thermal cyclers.

### Detection of EV infection in pancreatic tissue

A procedure developed in our laboratory was utilised to detect EV strains causing persistent infection [12]. Pancreas homogenates were cultured with a mix of EV-susceptible cell lines (AV3, RD, VC3, HEK-293) in order to enrich for virus (2–3 passages). To assess whether the infectious activity was linked to viral agents <100 nm in diameter, AV3 cells were incubated with culture supernatants and passed through 100 nm filters that remove cellular microorganisms and large viruses. RNA was extracted from supernatants of cells cultured in T25 flasks, then transcribed with Superscript III reverse transcriptase and VILO master mix. Five different EV-specific primer pairs were used for PCR assays in duplicate [12]). PCR tests were run on Verity Dx thermal cyclers. The LabChip GX Touch 24 capillary electrophoresis analyser was used to detect amplicons based on molecular size. A PCR assay was deemed positive when an amplicon peak of the expected size was observed in the electropherogram. PCR amplicons were sequenced by the Sanger method. Attempts were made to perform direct next-generation sequencing (NGS) of viral genomes by constructing Illumina libraries using cDNA prepared from the samples. These were, however, unsuccessful due to the extremely low quantities of virus-specific nucleic acids present in samples and the presence of overwhelming amounts of host nucleic acids.

### Detection of EV protein antigens in infected cell cultures

For IF, cell monolayers were prepared in Millicell EZ 4-well chamber slides (Merck) and fixed in PBS with 4% paraformaldehyde. Expression of EV protein antigens was tested by indirect IF using multiple pan-EV mAbs (ESM Table 1) directed to: (1) the VP1 capsid protein (mAbs 9D5, 6-E9/2, 5D8/1 [34]); (2) the 3D viral RNA polymerase (mAbs 3D-02 and 3D-05—our laboratory). Further controls included stainings with mAbs directed to coxsackieviruses group B; echoviruses 4, 6, 9, 11, 30, 34; echoviruses 4, 6, 9, 11, 30; and polioviruses 1–3. Alexa Fluor 488-goat anti-mouse IgG served as secondary antibody. Slides were counterstained with Evans blue. VP1 staining was deemed positive when granular cytoplasmic fluorescence was detected in infected cells. Staining for 3D RNA polymerase (3Dpol) typically produced speckled fluorescence in the nuclear area of persistently infected cells vs cytoplasmic staining of acutely infected cells. Images were taken with a Nikon E80i microscope and adjusted using Adobe Photoshop.

### Cell viability of the AV3 cell line exposed to virus isolates from pancreas

Cell monolayers grown in Millicell EZ 4-well slides were stained with the Live/Dead Viability Kit (Invitrogen) according to the manufacturer’s instructions. The assay discriminates live from dead cells by staining with green-fluorescent calcein-AM to indicate intracellular esterase activity and red-fluorescent ethidium homodimer-1 to indicate loss of plasma membrane integrity. Assays were run in duplicate and >800 cells/sample were counted by fluorescence microscopy. The percentage of dead cells was recorded and presented as percentage viability (mean ± SD).

### Release of cytokines by the AV3 cell line exposed to virus isolates from pancreas

Cytokine levels were measured in the medium of AV3 cells cultured in T25 flasks that had been incubated for 3 days with 0.25 ml of medium of EV-negative or of EV-positive cell cultures plus 4.75 ml of fresh medium. Each culture was then trypsinised and cultured again in fresh medium for 3 days. Cell culture supernatants were examined for 22 human cytokines using Luminex xMAP Technology assays (Myriad RBM) measuring granulocyte-macrophage colony-stimulating factor (GM-CSF), IFN-α, IFN-γ, IL-1-β, IL-2, IL-3, IL-4, IL-5, IL-6, IL-7, IL-8, IL-10, IL-12, IL-17, IL-18, macrophage inflammatory protein 1 (MIP1)-α, MIP1-β, monocyte chemoattractant protein-1 (MCP1), regulated on activation, normal T cell expressed and secreted (RANTES), monokine induced by gamma IFN (MIG), TNF-α and TNF-β.

### Statistics

Groups were compared using the unpaired two-tailed Student’s *t* test or the Mann–Whitney *U* test (GraphPad Prism). *p*<0.05 was considered statistically significant. No randomisation or blinding was carried out, and no data or samples were excluded.

## Results

### Detection of multiple viruses in the pancreas

Tables 1 and 2 list the investigated human pathogens (ordered according to their DNA [Table 1] or RNA [Table 2] genome). The results of nucleic acid assays revealed that EV genomes were present in pancreatic tissue of six of six diabetes cases of the DiViD study and also in two of 11 adenocarcinoma cases without diabetes. Viruses other than EVs were detected in only two individuals: EBV in the DiViD-5 case, and parvovirus B19 in the lateral pancreas nitrogen (LPN)-27 control. EBV and parvovirus B19 are known to establish persistent infections, especially manifest in morbid conditions. Thus, sporadic positivity for EBV or parvovirus is considered fortuitous. However, all virus-positive samples gave fluorescence signals in quantitative PCR tests only at high C_t_ values (C_t_ 28–34), indicating that minimal amounts of viral genomes were present. The possibility of detecting viruses in the pancreas has been confirmed by a study of ours showing that viral genomes are found in the presence of pancreatic enzymes when tissue is immediately frozen upon collection [35].

**Table 1:** Detection of multiple DNA viral agents in pancreatic tissue

Group	Case	DNA viruses									
		VZV	CMV	EBV	HHV6	HHV7	HHV8	BKV	JCV	Parvovirus B19	HBV
T1D cases at the clinical onset	DiViD-1	−	−	−	−	−	−	−	−	−	−
	DiViD-2	−	−	−	−	−	−	−	−	−	−
	DiViD-3	−	−	−	−	−	−	−	−	−	−
	DiViD-4	−	−	−	−	−	−	−	−	−	−
	DiViD-5	−	−	POS	−	−	−	−	−	−	−
	DiViD-6	−	−	−	−	−	−	−	−	−	−
Non-diabetic cases of pancreatic adenocarcinoma	LPN-01	−	−	−	−	−	−	−	−	−	−
	LPN-03	−	−	−	−	−	−	−	−	−	−
	LPN-08	−	−	−	−	−	−	−	−	−	−
	LPN-11	−	−	−	−	−	−	−	−	−	−
	LPN-14	−	−	−	−	−	−	−	−	−	−
	LPN-15	−	−	−	−	−	−	−	−	−	−
	LPN-17	−	−	−	−	−	−	−	−	−	−
	LPN-19	−	−	−	−	−	−	−	−	−	−
	LPN-21	−	−	−	−	−	−	−	−	−	−
	LPN-27	−	−	−	−	−	−	−	−	POS	−
	LPN-31	−	−	−	−	−	−	−	−	−	−

Data are from DiViD diabetes cases (*n*=6) and non-diabetic cases with pancreatic carcinoma (*n*=11)

BKV, BK polyomavirus; JCV, JC polyomavirus; POS, results of virus detection positive; T1D, type 1 diabetes; − indicates results of virus detection negative

**Table 2:** Detection of multiple RNA viral agents in pancreatic tissue

Group	Case	RNA viruses										
		HCV	HAV	Mumps	Rubella	Influenza A/B	Parainfluenza 1–4	RSV	Astrovirus	Norovirus	Rotavirus	EV
T1D cases at the clinical onset	DiViD-1	−	−	−	−	−	−	−	−	−	−	POS
	DiViD-2	−	−	−	−	−	−	−	−	−	−	POS
	DiViD-3	−	−	−	−	−	−	−	−	−	−	POS
	DiViD-4	−	−	−	−	−	−	−	−	−	−	POS
	DiViD-5	−	−	−	−	−	−	−	−	−	−	POS
	DiViD-6	−	−	−	−	−	−	−	−	−	−	POS
Non-diabetic cases of pancreatic adenocarcinoma	LPN-01	−	−	−	−	−	−	−	−	−	−	POS
	LPN-03	−	−	−	−	−	−	−	−	−	−	−
	LPN-08	−	−	−	−	−	−	−	−	−	−	−
	LPN-11	−	−	−	−	−	−	−	−	−	−	−
	LPN-14	−	−	−	−	−	−	−	−	−	−	−
	LPN-15	−	−	−	−	−	−	−	−	−	−	POS
	LPN-17	−	−	−	−	−	−	−	−	−	−	−
	LPN-19	−	−	−	−	−	−	−	−	−	−	−
	LPN-21	−	−	−	−	−	−	−	−	−	−	−
	LPN-27	−	−	−	−	−	−	−	−	−	−	−
	LPN-31	−	−	−	−	−	−	−	−	−	−	−

Data are from DiViD diabetes cases (*n*=6) and non-diabetic cases with pancreatic carcinoma (*n*=11)

EV, EVs of the A to D species (over 100 types); HAV, hepatitis A virus in the family Picornaviridae; Influenza A/B, influenza virus types A and B; Mumps, mumps virus; Parainfluenza 1–4, parainfluenza virus types 1–4; POS, results of virus detection positive; Rubella, rubella virus; T1D, type 1 diabetes; − indicates results of virus detection negative

### Detection of EVs in pancreas using virus enrichment in cell cultures followed by molecular tests

To confirm EVs’ presence in the pancreas and to assess EV infectivity, we incubated tissue homogenates with cultures of human cells for 3–5 days. Cell cultures were subjected to 2–3 serial passages, then five different PCR assays to the enteroviral 5′ untranslated region (5′UTR) region were run on retrotranscribed RNA. The search for EV genomes gave positive results in six of six DiViD cases and in two of 11 LPN control cases (LPN-01 and LPN-15). As shown in Table 3, partial sequences of the 5′UTR or the VP4–VP2 regions confirmed the EV genomes in six DiViD cases and in the two LPN control cases. The detected viral sequences were compatible with infection due to members of the enteroviral B species, with the exception of DiViD-4 which was compatible with members of the EV-C species, and LPN-15 which was compatible with members of the EV-A species. EV sequences showed 94–98% identities with deposited enteroviral sequences and no similarities with sequences of other viruses or human genes.

**Table 3:** Partial RNA genome sequences of EV strains obtained from the pancreases of DiViD diabetes cases and non-diabetic cases of pancreatic carcinoma

Group	Case	Sequence	Genome region	Matching EV species^a^	Compatible EV type	Identities	Gaps
T1D cases at the clinical onset	DiViD-1	GCGGTTGAAGGAGAAAGCGTTCGTTATCCGGCCAACTACTTCGAAAAACCTAGTAACACCGTGGAAGTTGCAGAGTGTTTCGCTCAGCACTACCCCAGTGTAGATCAGGTCGCTGAGTCACCGCATTCCCCACGGGCGACCGTGGCGGTGGCTGCGTTGGCGGCCTGCCCATGGGGGAACCCATGGGACGCTCTAATACAGACATGGTGCGCAGAGTCTATTGAGCTAGTTGGTTGTCCTCCGGCGCCTGAATGCGGCTAATCCTAACTGCGGAGCACACACCCTCAAGCCAGAGGGCAGTGTGTCGTAACGGGCAACTCTGCAGCGGAACCGACTACTTTGGGTGTCAGTGTTTCATTTTATTCATATACTGGCTGCTTATGTTG	5’UTR	B	Coxsackievirus B3	380/387	1
		CTAACTATCGGGACAGGACAGCCGCAACCCCAGTGGGCAGTCTGTCGTAACGGGTAAGCTCTGTCAGCGGAACCGACTACTTTGGTGTCCGTGTTTCAATAATGACCGTGTTTCAAGAAAAAAACCAAACCCAAAAACAAGCAAAACAAAACCCCCCCACCCCACCCC	VP4-VP2	B	EV B	75/82	4
	DiViD-2	GAGAAAACGTTCGTTACCCGGCCAACTACTTCGAAAACCCTAGTAACACCATGGAAGTTGCAGAGTGTTTCGCTCAGCACTACCCCAGTGTAGATCAGGTCGATGAGTCACGGCATTCCCCACGGGCGACCGTGGCGGTGGCTGCGTTGGCGGCCTGCCGATGGGGAAACCCATAGGACGCTCTAATACAGACATGGTGCGAAGAGTCTATTGAGCTAGTTGGTAGTCCTCCGGCCCATGAGTGCGGCTAATCCTAACTGCGGAGCACACACCCTCAAGCCAGGGGGCAGTGTGTCGTAACGGGCAACTCTGCAGCGGACCCGACTACTTTGGGTGTCCGTGTTTCCCCTTACTCTTGTATTGGCTG	5’UTR	B	Echovirus-17	361/367	0
	DiViD-3	TGAGTCACCGCATTCCCCACACCCGACTGTGGCGGTGGCTGCGTTGGCGGCCTGCCCATGGGGCAACCCATGGGACGTTTCAATACTGACATGGTGCGAAGAGTCTATTGAGCTAATTGGTAGTCCTCCGGCCCCTGAATGCGGCTAATCGAATCTGCGGAGCAGGCACTCGCAGACCAGCGAGCAGCTTGTSGTAATGGGCAACTCCGCAGGGAAGCGACTACTTTGGGTGACCGTGTTTCCTAT	5’UTR	B	Coxsackievirus B6	239/247	1
	DiViD-4	GGTGACGGTGGTCCAGGCTGCGTTGGCGGCCTACCTGTGGCCCACAGCCACAGGACGCTAGTTGTGAACAAGGTGTGAAGAGGGTATTGAGCTACAAGAGAGTCCTCCGGCCCCTGTATGCGGCTAATCCCAACCACGGAGCAAGGGTACACAAGCCAGTGTATACCTTGTCGTAACGCGCAAGTCTGTGGCGGAACCGACTACTTTAGGTGTCCGTGTTTCC	5’UTR	C	Coxsackievirus A13	218/223	0
	DiViD-5	AATTACTTCGAGAAACCTAGTAACACCATGAAAGTTGCGTAGTGTTTCGCTCCGCACAACCCCAGTGTAGTTTAGGTCGATGAGTCACCGCAATCCCCACGGGCGACCGTGGCGGTGGCTGCGTTGGCGGCCTGCCCATGGGGCAACCCATGGGACGCTTCAATACTGACATGGTGTGAAGAGTCTATTGAGCTAATTGGTCCTCCTCCGGCCCTGAATGCGGCTAATCCCAACTGCGGAGCAGACGCTCGCATGCCAGCGAGTAGTCTGTCGTAACGGGCATCTCTGCAGCGGAAC	5’UTR	B	Echovirus-18	292/298	1
	DiViD-6	CTTCTGTTACCCCGGCTGATATCAATAAGCTGCTCACGTGGCTGAAGGAGAAAACGTTCGTTATCCGGCCAATTACTTCGAGAAACCCAGTACCACCATGAAAGTTGCGCGGCGTTTCGCTCCGCACAACCCCAGTGTAGATCGGGCCGATGAGTCACCGCGTTCCCCACGGGCGACCGTGGCGGTGGCTGCGTTGGCGGCCTGCCCATGGAGCAATCCATGGGACGCTTCAATACTGACATGGTGCGAAGAGTCTATTGAGCTAATTGGTAGTCCTCCGGCCCCTGAATGCGGCTAATCCTAACTGCGGAGCAGATACCCACACGCCAGTGGGCAGTCTGTCGTAACGGGCAACTTCGCAGCGGAACCGACTACTTTGGGTGTCCGTGTTTCCTTTTATTTTATACTGGCTGCTTATGGTGACAATCA	VP4-VP2	B	Echovirus-30	425/429	1
Non-diabetic cases of pancreatic adenocarcinoma	LPN-01	TTTTGATCAAGCACTTCTGTTACCCCGGACTGAGTATCAATAGACCGCTAACGCGGTTGAAGGAGAAAACGTTCGTTACCCGGTCAACTACTTCGAAAAACCTAGTAACACCATGGAAGTTGCGGAGTGTTTCGCTCAGCACTACCCCAGTGTAGATCAGGTCGATGAGTCACCGCGTTCCCCACGGGCGACCGTGGCGGTGGCTGCGTTGGCGGCCTGCCTACGGCGAAACCCGTAGGACGCTCTAATACAGACATGGTGCGAAGAGTCTATTGAGCTAGTTGGTAATCCTCCGGGACCCTGAATGCGGCTAATCCTAACTGCGGAGCACATACCCTCAAACCAGGGGGCAGTGTGTCGTAACGGGCAACTCTGCAGCGGAACCGACTACTTTGGGTGTCCG	5’UTR	B	Coxsackievirus B1	399/403	1
	LPN-15	AAGGAGAAAACGTTCGTTATCCGGCTAACTACTTCGAGAAACCTAGTCGCACCATGAAAGTTGCGGAGTGTTTCGCTCATCCCTTCCCCCGTGTAGATCAGGTCGATGAGTCACTGCATTCCCCACGGGCGACCGTGGCAGTGGCTCGTTGGCGGCCTGCCTATGGGGTAACCCATAGGACGCTCTAATACGGACATGGTGCGAAGAGTCATTGAGCTAGTTAGTAGTCCTCCGGCCCCTGAATGCGGCTAATCCTAACTGCGGAGCACATACCTTCAATCCAGGGGGCGGTGTGTCGTAATGGGCAACTCTGCAGCGGAACCGACTACTTTGGGTGTCTGTGTTTCCTTTTATTCTTATATTGG	5’UTR	A	Coxsackievirus A2	360/367	2

^a^EV sequences were analysed with the public databases listed in ESM Table 1

T1D, type 1 diabetes

To assess the EV type of viral strains present in pancreases, we attempted to amplify the regions coding for the enteroviral capsid protein VP1 via NGS and other methods reviewed by Cassidy et al [36]. Our efforts were unsuccessful, presumably because of the minimal virus load combined with the huge variability of the capsid-coding regions of >240 different EV types. Similarly, attempts to perform NGS with Illumina libraries failed due to the extremely low load of viruses. Of note, the DiViD-6 case, which had the highest virus load, was attributed to echovirus-30 in total agreement with the results of Oikarinen et al [29]. In this case, IHC revealed VP1-positive cells not only in islets but also in ductal cells, indicating an infection more widespread as compared with that seen in DiViD cases 1 to 5 (data not shown).

### Detection of EV-coded protein antigens in the AV3 cell line incubated with filtered cell culture supernatants

**Fig. 1:**
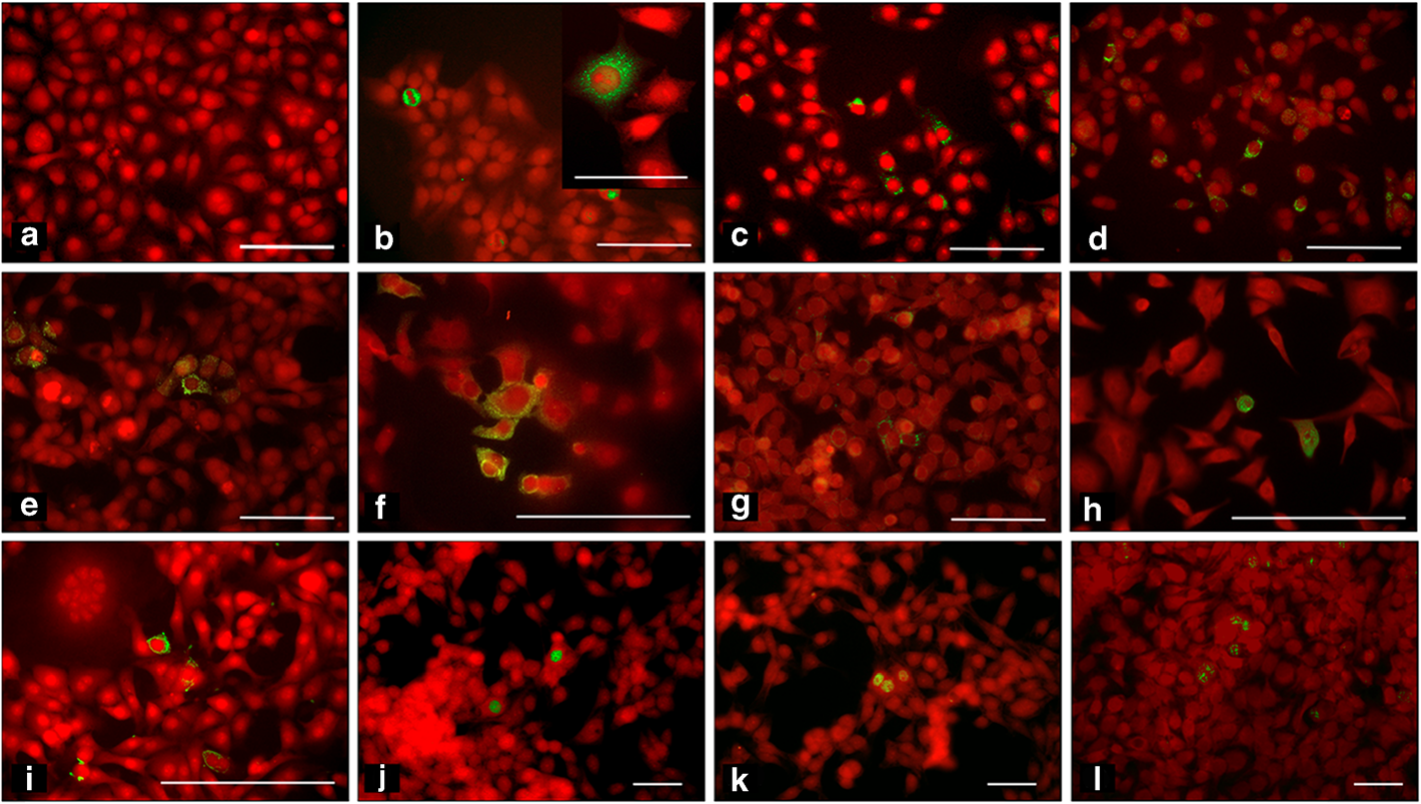
Immunofluorescence staining of cultured cell monolayers infected with EV strains (green) isolated from the diabetic DiViD cases and the pancreatic adenocarcinoma cases without diabetes. Counterstaining with Evans blue (red). (**a**–**i**) Cytoplasmic staining of the enteroviral capsid protein using mAb 6-E9/2: (**a**) negative control: uninfected AV3 cell monolayer. DiViD cases: DiViD-1 (**b**; inset shows the granular pattern of cytoplasmic staining); DiViD-2 (**c**); DiViD-5 (**d**); DiViD-6 [(**e**) infection before filtration; (**f**) second passage after filtration through 100 nm membrane]. LPN cases without diabetes: LPN-01 [(**g**) infection before filtration; (**h**) second passage after filtration through 100 nm membrane]; LPN-15 (**i**). (**l**–**n**) Staining by mAb 3D-05 to the enteroviral RNA polymerase enzyme (speckled nuclear staining): DiViD-1 (**j**), DiViD-2 (**k**), DiViD-6 (**l**). Scale bars represent 100 μm or 50 μm in the higher-magnification picture (**b**, inset)

To define the infectious nature of virus strains isolated from pancreas, supernatants of EV-positive DiViD and LPN samples were sieved through 100 nm filters (i.e. filters excluding bacteria and also viruses >100 nm in size). Cultures of the AV3 cell line were incubated with filtered samples (1:10 [vol/vol] dilution). Three days later, cell monolayers were fixed with paraformaldehyde and stained with mAbs to enteroviral capsid protein VP1 and the RNA polymerase enzyme (3Dpol). Multiple reagents were used to confirm the enteroviral nature of the detected agents. As shown in Fig. 1, mAbs to VP1 identified the infected cells expressing the VP1 capsid protein. The percentage of VP1-expressing cells in virus-positive cell cultures has been evaluated by IF: 1.46 ± 0.57 (mean ± SD; range 0.6-4.7; *n*=14). Granular fluorescence staining was located in the cytoplasm (Fig. 1b–h). Three independent VP1 antibodies, 9D5, 6-E9/2 and 5D8/1, gave comparable results. For all viral isolates, infectivity could be passed in series from one culture to a new one after filtration through 100 nm membranes, demonstrating the presence of a virus smaller than 100 nm, a size compatible with the 30 nm diameter of EVs (Fig. 1f,h).

When cell monolayers were stained for the EV-encoded 3Dpol enzyme using mAbs produced in our laboratory, we observed speckled fluorescence in the nucleus (Fig. 1j,k,l). It should be noted that in acute infection, speckled 3Dpol fluorescence is situated in the cytoplasm (A. Toniolo, unpublished observations), while persistent infection is characterised by nuclear localisation. The nuclear staining supports a persistent mode of infection.

### IHC of EV-positive cases

**Fig. 2:**
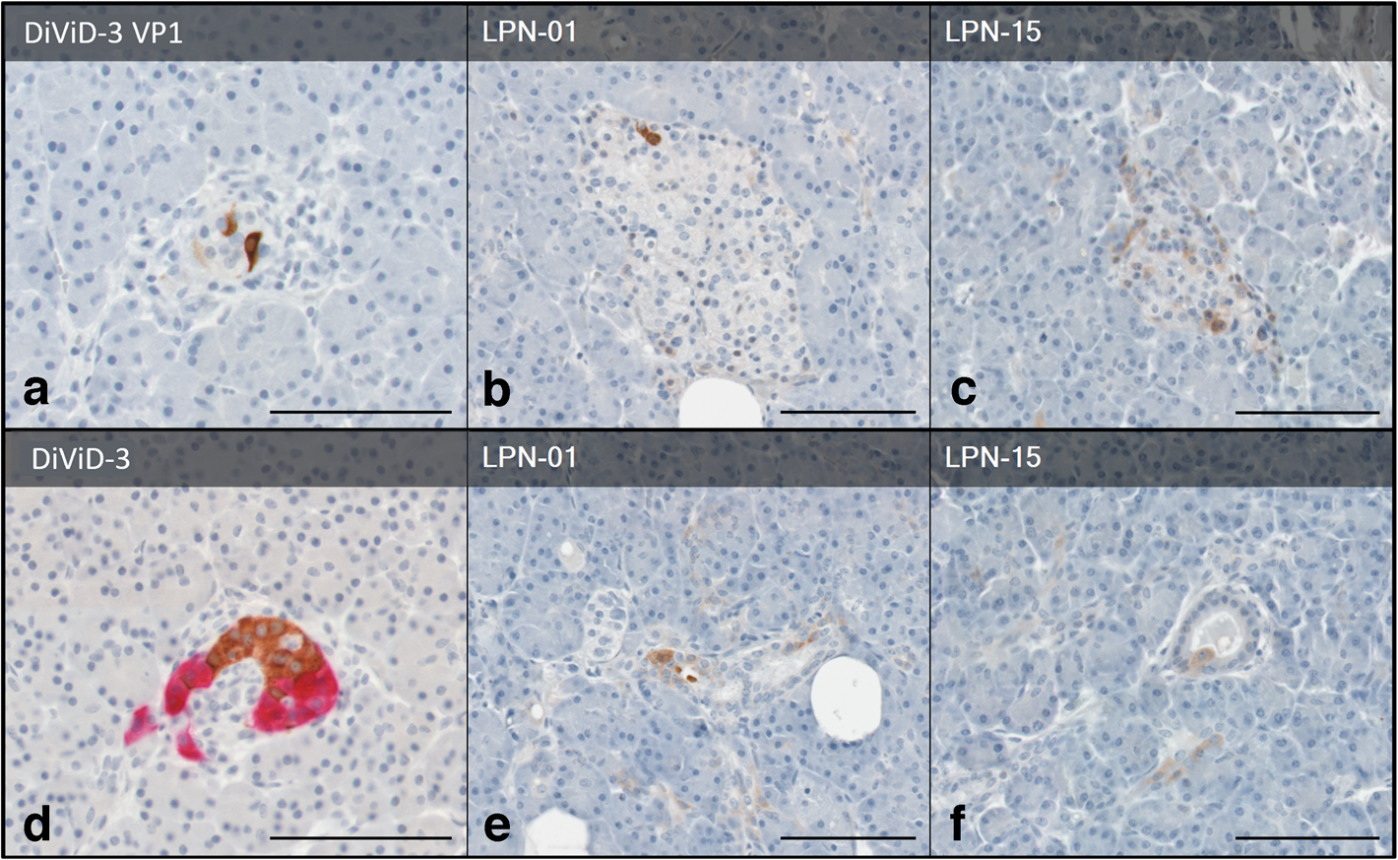
Representative immunostaining of enteroviral VP1 (brown) in pancreas tissue from (**a**) DiViD donor 3 (DiViD-3), and two pancreas resections from donors without diabetes, (**b**) LPN-01 and (**c**) LPN-15. All donors tested positive for enteroviral PCR. (**d**) The serial section of DiViD-3 is immunostained with insulin (brown) and glucagon (red), confirming this is an insulin-containing islet. Occasional ductal VP1^+^ cells were identified in (**e**) LPN-01 and (**f**) LPN-15. All sections are counterstained with haematoxylin. Scale bars, 100 μm

Pancreas sections of EV-positive cases (both DiViD and LPN) were stained for insulin and for enteroviral VP1 to assess the sites of viral infection. Fig. 2 shows that insulin-positive islets of the DiViD-3 case contained a few VP1-positive cells (Fig. 2a), together with reduced insulin content in selected islets (Fig. 2d). Similarly, tissue of LPN-01 and LPN-15 cases had few VP1-positive islet cells (Fig. 2b,c), but VP1 was also observed in ductal cells, indicating that the acinar tissue was also involved in infection (Fig. 2e,f).

### Exposure of the AV3 cell line to EV-positive and EV-negative supernatants of DiViD and LPN cases: effects on cell viability and on cytokine production

To explore the possible biological effects of the EV isolates obtained from DiViD and LPN cases, the AV3 cell line was incubated with a 1:20 dilution of culture supernatants derived from the DiViD and LPN cases. Three days later, cells were trypsinised and re-grown in fresh medium for 3 days. Cell viability and the production of different cytokines were assessed.

**Fig. 3:**
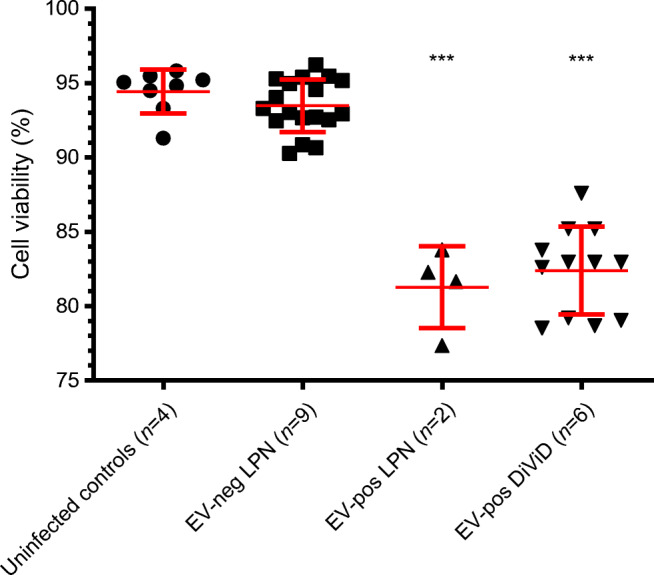
Percentage viability of cultured AV3 cells either uninfected or exposed to: EV-negative or EV-positive LPN supernatants, or six of six EV-positive DiViD supernatants. Three days post-treatment, each sample was tested in duplicate using a live/dead fluorescence assay. Results are presented as scatterplots with mean ± SD. The percentage viability of cultures incubated with virus-infected samples was significantly lower (***p<0.001) compared with that of uninfected controls and of cultures exposed to EV-negative LPN supernatants. neg, negative; pos, positive

Figure 3 shows that, compared with uninfected control cells and with cells incubated with EV-negative supernatants of LPN cases, viability of cells exposed to EV-positive supernatants (LPN or DiViD cases) was moderately reduced (80–85% that of controls; *p*<0.001). This helps in explaining why such modest changes escaped detection by microscopy and were previously seen only using time-lapse microscopy [12].

**Fig. 4:**
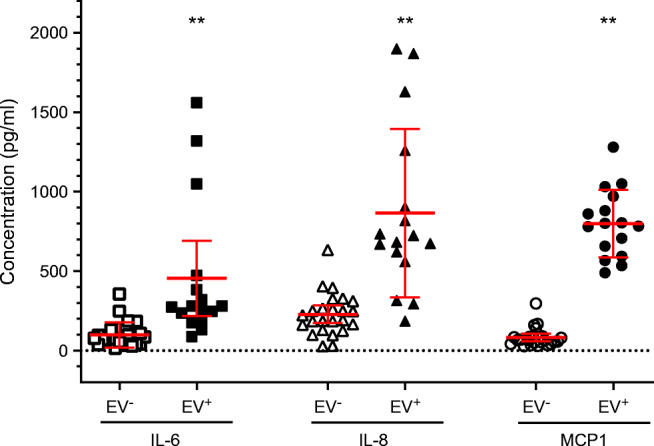
Cytokine levels in culture medium of AV3 cells that had been incubated with 0.25 ml of culture medium from EV-negative or EV-positive cases (cells were cultured in T25 flasks containing 5 ml of medium for 3 days, trypsinised and cultured again with fresh medium for 3 days). Each supernatant was tested in duplicate for a panel of 22 cytokines using Luminex xMAP Technology assays. Each symbol represents a single measurement. Results are shown as scatterplots with mean ± SD. Compared with cultures exposed to virus-negative supernatants, those exposed to EV-positive supernatants produced significantly enhanced levels of IL-6, IL-8 and MCP1 (**p<0.01), but not of other cytokines

The levels of cytokines released by AV3 cells incubated with EV-positive supernatants of DiViD and LPN cases were compared with those of uninfected cells or of cells exposed to EV-negative supernatants of LPN cases. Twenty-two cytokines/growth factors were tested. Significant differences were only obtained for IL-6, IL-8 and MCP1 (all *p*<0.001). The scatterplot of Fig. 4 shows the levels of differentially produced cytokines: higher levels in cultures incubated with EV-positive supernatants (range of medians 277–792 pg/ml) vs lower levels in cultures incubated with supernatants of EV-negative cases (range of medians 66-207 pg/ml).

## Discussion

Mounting evidence shows that viral infections play a role in type 1 diabetes and other immune-mediated conditions [15, 37–40]. At the time of clinical onset, blood leucocytes of children/adolescents frequently carry EVs [41] and these agents are also present in spleen and lymph nodes of organ donors with diabetes independently of diabetes duration (unpublished data—nPOD-V consortium, https://www.jdrfnpod.org/publications/npod-working-groups/npod-viral-work-group/). Close to the time of type 1 diabetes onset, EVs are frequently spreading among family members of probands [42]. EV infection occurs more frequently in children/adolescents with insufficient levels of vitamin D [43]. In addition, histopathology demonstrated that islet cells contain EV antigens, and the presence of virus is concomitant with islet alterations that include reduced insulin content, expression of HLA class I antigens and expression of ISGs [3]. Longitudinal studies showed that EVs are present in stools of children predisposed to type 1 diabetes before the development of pancreatic autoimmunity [9]. Studies conducted with advanced sequencing methods concluded that EVs were predominant in stools during the period before debut of type 1 diabetes [10, 44].

The first report of the DiViD study demonstrated the EV VP1 protein in pancreases of all six cases and the EV genome in one of six cases. In enriched islets, enteroviral genome was found in the culture medium of four of six cases [4]. Using highly sensitive methods, we demonstrate that EV genomes are present in the pancreases of all six cases. This finding is in line with earlier studies demonstrating EV genomes in PBMCs, duodenal biopsies or stool of all six DiViD cases [29].

A major aim of our study was to determine whether infections by viral agents other than EVs could contribute to diabetes. We demonstrate that: (1) viral agents other than EVs are hardly detected in pancreas; (2) in cases of newly diagnosed type 1 diabetes, EVs are consistently detected in pancreas, whereas in pancreatic carcinoma detection of EVs is an occasional finding; (3) EV strains obtained from both DiViD cases and LPN control cases are capable of growth in cultured cells; (4) the EV strains pass through 100 nm membranes and can be serially passaged in cell cultures; (5) upon infection with the isolated strains, cultured cells express EV-coded proteins (the VP1 capsid protein and the 3Dpol enzyme), showing that the infecting viruses are viable; (6) infection modestly reduces the viability of cultured cells in ways consistent with low-grade viral replication; (7) infection of cultured cells stimulates the release of some cytokines; (8) pancreas IHC confirmed that EV VP1 is expressed in the islets of Langerhans and, in some cases, in the acinar tissue of virus-positive DiViD and control cases.

It is of interest that HHV-6 and parvovirus B19, two viral agents prevalent in the thyroid tissue [45] could not be detected in the pancreas. This confirms the specific pancreatic tropism of EVs [46] and that their link with type 1 diabetes is not by chance. Hence, the EV genus is viewed as a possible ‘diabetogenic’ group of viral agents.

Partial RNA sequencing showed that the detected EV strains represent members of the B, A and C species: (1) partial genome sequences demonstrate that different cases were associated with independent viruses, thus rejecting the risk of laboratory contamination and the hypothesis that a single EV type can be linked to diabetes; (2) staining of viral antigens with mAbs of different specificities for the EV genus robustly supports the findings; (3) in agreement with results of Oikarinen et al [29], the DiViD-6 case, which had the highest virus load, was recognised as echovirus-30 through sequencing of the VP4–VP2 capsid region. IHC of DiViD-6 showed that VP1-positive cells were present not only in the islets but also in ductal cells of the acinar tissue, suggesting a more widespread infection compared with that observed in DiViD cases 1–5.

The data show that EVs of different species and/or types are linked to the six DiViD cases. This, if confirmed by further studies, may indicate the need for a multivalent (not monovalent) enteroviral vaccine for reducing the risk of type 1 diabetes [38, 47].

A limitation of the study is that the cases without diabetes used as control cases were older than type 1 diabetes cases. Thus, the prevalence of pancreatic EV infection remains unknown in younger populations. In addition, diabetes-related autoantibodies and HLA typing were not available for control cases. Future research should investigate pancreatic tissue from individuals of younger ages to elucidate whether sporadic EV positivity also occurs in the young.

A further limitation is that the methods used in the study did not allow us to generate VP1 sequences required to identify individual EV types. In type 1 diabetes cases and control cases, this was likely due to the nature of the involved agents: mutated viruses capable of very low-level replication in vitro and accumulating only tiny amounts of genome in infected tissues. Previous literature concluded that persistent EV infections were unlikely in chronic human disease but also stated that proof of persistence would be satisfactory if evidence for the contemporary presence of viral genome and the encoded proteins was presented [48]. Recently, the characterisation of chronic EV infection in a severely ill patient demonstrated that chronic EV infection does occur, that it may produce severe pathology, that the involved EV strains are highly mutated and that full sequences of chronic EV strains are difficult to obtain even by using the most advanced techniques [36]. Our study demonstrates that viral genomes and their encoded proteins can be detected both in pancreas tissue and in cultured cells, and, importantly, that EV strains obtained from pancreas are alive and infectious. Thus, these strains are not remnants of inert nucleic acids.

The possibility of typing the EV types linked to type 1 diabetes depends on the development of more sensitive sequencing tools [10, 36] capable of obtaining complete de novo assemblies of viral genomes from infected patients (or cultured cells exposed to patients’ tissues) and to resolve viral mixtures into multiple variants. In addition, novel high-throughput serology methods may help in indicating that EVs are associated with type 1 diabetes [49]. The above approaches will allow recognition of the EV types causing/triggering diabetes across different populations. Studies of this kind pave the way to the possible prevention of type 1 diabetes by vaccines and to treatment of early-stage diseases with antiviral drugs [50].

In closing, our results confirm that EV genome and capsid proteins are consistently present in the pancreases of newly diagnosed diabetes cases but rarely found in the pancreases of individuals without diabetes. Essentially, no other viruses were found in the investigated cases. Finally, EV infections could be transmitted from EV-positive cell cultures to uninfected cultures, indicating that the viral agents detected in the early phase of type 1 diabetes are infectious. This may help explain the spread of type 1 diabetes among family members [42] since, in people with diabetes, low levels of infectious virus are present in blood and stools, two well-recognised vehicles of infection within families.

## Supplementary Information

ESM (PDF 234 kb)

## Data Availability

The datasets presented in this article are not readily available because of the sensitive nature of the data and possible high risks associated with patient confidentiality. Requests to access the datasets should be directed to lars.krogvold@odont.uio.no.

## Abbreviations

3Dpol: 3D RNA polymerase
5′UTR: 5′ Untranslated region
CBV: Coxsackievirus B
CMV: Cytomegalovirus
DiViD: Diabetes Virus Detection
EBV: Epstein–Barr virus
EV: Enterovirus
FFPE: Formalin-fixed paraffin-embedded
HAV: Hepatitis A virus
HBV: Hepatitis B virus
HCV: Hepatitis C virus
HHV: Human herpesvirus
IF: Immunofluorescence
IHC: Immunohistochemistry
ISG: IFN-stimulated gene
LPN: Lateral pancreas nitrogen
mAb: Monoclonal antibody
MCP1: Monocyte chemoattractant protein-1
MIP1: Macrophage inflammatory protein 1
NGS: Next-generation sequencing
PBMC: Peripheral blood mononuclear cell
RSV: Respiratory syncytial virus
VZV: Varicella-zoster virus
