# Supplementary material for: Live enteroviruses, but not other viruses, detected in human pancreas at the onset of type 1 diabetes in the DiViD study
Source: Diabetologia. 2022 Aug 12;65(12):2108–20. doi: 10.1007/s00125-022-05779-2 (PMC9630231; doi:10.1007/s00125-022-05779-2)
Supplement: Supplementary file 1 — (PDF 234 kb) [file 125_2022_5779_MOESM1_ESM.pdf]

SUPPLEMENTARY MATERIALS

THE DiViD STUDY - LIVE ENTEROVIRUSES, NOT OTHER VIRUSES, DETECTED IN HUMAN PANCREAS AT THE ONSET OF TYPE 1 DIABETES

Lars Krogvold[1,2], Angelo Genoni [3], Anna Puggioni [3], Daniela Campani [4], Sarah. J Richardson [5], Christine S. Flaxman [5], Bjørn Edwin [6], Trond Buanes [6], Knut Dahl-Jørgensen [1], Antonio Toniolo [7].

1. Division of Pediatric and Adolescent Medicine, Oslo University Hospital, Oslo, Norway
2. Institute of Clinical Dentistry, Faculty of Dentistry, University of Oslo, Norway
3. Department of Biotechnology and Life Sciences, University of Insubria, Varese, Italy - angelopaolo.genoni@uninsubria.it – ORCID ID 0000-0001-9848-2981
4. Department of Oncology, University of Pisa, Pisa, Italy – daniela.campani@unipi.it - ORCID ID 0000-0001-5987-734X
5. Islet Biology Group (IBEx), Exeter Centre of Excellence in Diabetes (EXCEED), University of Exeter College of Medicine and Health, Exeter EX2 5DW, UK.
6. Department for HPB surgery, Oslo University Hospital, Oslo, Norway
7. Global Virus Network, University of Insubria, Varese, Italy - – antonio.toniolo@uninsubria.it – ORCID ID 0000-0003-3008-2126

ESM TABLE 1

ESM Table 1. List of consumables, chemicals, cell lines, viruses, culture media, antibodies, reagents for molecular biology, commercial PCR kits, instruments, virus databases, software.

Resource or Reagent	Obtained from
**Blood test tubes**	
Vacutainer K_2EDTA tube, Vacutainer K_2EDTA Plasma Preparation tube	Becton Dickinson (BD, Milano, Italy)
**Plasticware**	
Flasks (T-25; T-75); 12-well and 6-well multiplates; cell scrapers; pipettes	Thermo Fisher Scientific (Monza, Italy)
Millicell EZ 4-well glass slides; Millex® filter units PVDF pore size 220 nm, 100 nm	Merck-Millipore (Vimodrone, Italy)
**Cell culture**	
Cell lines AV3, RD, VC3, HEK-293	European Collection of Authenticated Cell Cultures (ECACC, Salisbury, UK)
Cell line LLC-MK2; Coxsackievirus types B1-B6	American Type Culture Collection (ATCC, Manassas, VA)
Fetal bovine serum (FBS); DME/F12 medium with Hepes; L-Glutamine; Pyruvate; Penicillin-Streptomycin; Hank's balanced salt solution (HBSS); Dulbecco Phosphate-buffered saline (DPBS); Trypsin-EDTA	LifeTechnologies-Gibco (Monza, Italy)
Histopaque cell separation medium (density 1.077 and 1.119 g/ml); Bovine serum albumin (BSA); Collagenase type IV; Dispase-I	Sigma-Aldrich (Milano, Italy)
PANTA antibiotic mixture (Polymyxin B; Amphotericin B; Nalidixic Acid; Trimethoprin; Azlocillin)	Becton Dickinson (BD, Milano, Italy)
MycoAlert Plus Mycoplasma Detection Kit; Accutase (cell detachment enzymes)	Euroclone-Lonza (Pero, Italy)
Live/Dead™ Viability/Cytotoxicity Kit for mammalian cells	Thermo Fisher Scientific (Monza, Italy)
**Molecular biology**	
GoTaq DNA hot start DNA polymerase and master mix; DNA molecular weight markers; Tris-Acetate EDTA buffer (TAE); Agarose; High-resolution agarose	Promega Italia (Milano, Italy)
Reagents and disposables for the m2000sp automated instrument; DNA and RNA preparation kits; reagent vessels; deep well plates; disposable tips	Abbott Molecular (Rome, Italy)
Custom oligonucleotide primers; Water (PCR-grade); Tris-EDTA buffer pH 8.0 (TE); low-EDTA (0.1 mM) TE buffer pH 8.0; Elution (10 mM Tris-Cl) buffer EB pH 8.5	Sigma-Aldrich (Milano, Italy)
Superscript III and Superscript IV reverse transcriptase with VILO master mix [containing ribonuclease inhibitor, helper and stabilizer proteins, random hexamer primers OR mixture of random hexamer primers plus oligo (dT)18; dNTPs; $MgCl_2$]; Platinum Taq hot start DNA polymerase and PCR Master Mix; Platinum GC Enhancer	Thermo Fisher Scientific (Monza, Italy)
Brilliant II SYBR QPCR Master Mix with ROX passive reference dye	Agilent Technologies (Cernusco sul Naviglio, Italy)
GelRed stain	DBA Italia-Biotium (Segrate, Italy)
FlashGel - DNA electrophoresis screening system	Euroclone-Lonza (Pero, Italy)
BigDye Terminator V1.1 Cycle Sequencing Kit; Centri-Spin purification columns	Thermo Fisher Scientific (Monza, Italy)
LabChip GX Touch 24 Nucleic Acid Analyzer; HT DNA HS Reagents kit dual protocol; Hardshell PCR plate-96 blue	Perkin Elmer Italia (Milano, Italy)
Xpose spectrophotometer	Trinean (Gentbrugge, Belgium)
ABI Prism 7500 thermocycler; Verity Dx thermocycler	Thermo Fisher Scientific (Monza, Italy)
**Chemicals (molecular biology grade)**	
Ethanol; Isopropanol; Dimethyl sulfoxide (DMSO); N,N-Dimethylformamide; Acetone; Paraformaldehyde 16% ampules (PFA); Evans Blue; Triton X100; Tween-20; Na-Azide	Sigma-Aldrich (Milano, Italy)
**Commercial PCR kits for virus detection**	

HBV, HCV, CMV, EBV, BK polyomavirus	Abbott Molecular (Roma, Italy)
VZV, HHV6 A and B, JC polyomavirus, parvovirus B19, influenza virus A and B, parainfluenza virus 1-4, RSV A and B, astrovirus species 1-8, norovirus genogroup I and II, rotavirus, HAV, enteroviruses	Altona Diagnostics Italia (Segrate, Italy)
HHV7	Sacace Biotechnologies (Como, Italy)
Rubella virus	Bioron Diagnostics (Römerberg, Germany)
Mumps virus	MyBiosource Gentaur (Bergamo, Italy)
**Antibodies to enteroviruses; indirect immunofluorescence**	
Mouse monoclonal antibodies (MAbs): 9D5 (panenterovirus directed to the VP1 capsid protein); Coxsackie A9; Coxsackie A24 (cross reacting with Echovirus 34); Coxsackie B Blend; Coxsackie B1; Coxsackie B2; Coxsackie B3; Coxsackie B4; Coxsackie B5; Coxsackie B6; Echovirus Blend 4, 6, 9, 11, 30, 34 (cross reacting with Coxsackie A24); Echovirus 4; Echovirus 6; Echovirus 9; Echovirus 11; Echovirus 30; Enterovirus 70; Enterovirus 71 (cross reacting with Coxsackie A16); Poliovirus Blend; Poliovirus-1; Poliovirus-2; Poliovirus-3.	Merck-Millipore (Vimodrone, Italy)
Mouse MAb 6-E9/2 "Magic" (panenterovirus directed to the VP1 capsid protein)	Creative Diagnostics (Shirley, NY)
Mouse MAb 5D-8.1 (panenterovirus directed to the VP1 capsid protein)	Dako (Milano, Italy)
Mouse MAbs 3D-02 and 3D-05 (panenterovirus directed to the 3Dpol enzyme)	Our own laboratory
Alexa Fluor 488 goat anti-mouse IgG; FITC goat anti-rabbit IgG; FITC rabbit anti-goat IgG; ProLong antifade; Evans Blue; DAPI	Thermo Fisher Scientific (Monza, Italy)
**Cytokine levels in supernatants of cultured cells**	
Luminex xMAP Technology assay - human cytokines: GM-GSF, IFN-alpha, IFN-gamma, IL1-beta, IL2, IL3, IL4, IL5, IL6, IL7, IL8, IL10, IL12, IL17, IL18, MIP-1-alpha, MIP-1-beta, MCP-1, CCL5/RANTES, MIG, TNF-alpha, TNF-beta	Myriad RBM (Austin, TX) through DBA Italia (Milano, Italy)
**Immunofluorescence microscopy**	
Nikon E80i microscope with 20x and 40x IF objectives	Nikon (Firenze, Italy)
**Databases and software (virus sequences, statistics, graphics)**	
National Library of Medicine Viral Genomes, https://www.ncbi.nlm.nih.gov/genome/viruses/	NLM Viral genomes (USA) - last accessed: 2022-05-03
Reference Viral DataBase (RVDB), https://rvdb.dbi.udel.edu/	Reference Viral Database, RVDB (USA)- last accessed: 2022-04-28
Virus Pathogen Resource (ViPR), https://www.viprbrc.org/brc/home.spg?decorator=vipr	Virus Pathogen Resource, ViPR (USA) - last accessed: 2022-04-12
ViralZone is a SIB Swiss Institute of Bioinformatics web-resource, https://www.re3data.org/repository/r3d100013314	Swiss Institute of Bioinformatics, SIB (Switzerland) – last accessed: 2022-04-29
GraphPad Prism v. 8	GraphPad Software, San Diego, CA

ESM Table 2. Newly diagnosed cases of type 1 diabetes and non-diabetic cases of pancreatic adenocarcinoma.

Group	Case	Age, years	Sex	Weeks from T1D diagnosis until biopsy	Surgical treatment	Insulin treatment (U/Kg/day)	Anti-GAD (<0.08 au[1])	Anti-insulin (<0.08 au)	anti-ZnT8 (<0.12 au)	anti-IA2 (<0.10 au)	HLA risk alleles[2]
T1D cases at the clinical onset	DiViD-1	25	F	4	Laparoscopy (LAP)	0.50	1.76	0.7	0.28	0.16	Yes
	DiViD-2	24	M	3	LAP	0.35	0.79	<0.01	0.44	>3	Yes
	DiViD-3	34	F	9	LAP	0.17	1.77	< 0.05	1,.45	> 3	Yes
	DiViD-4	31	M	5	LAP	0.40	0.77	0.1	< 0.01	2.54	Yes
	DiViD-5	24	F	5	LAP	0.36	0.46	0.1	0.06	>3	Yes
	DiViD-6	35	M	5	LAP	0.52	1.85	< 0.05	< 0.01	< 0.04	Yes
Non-diabetic cases of pancreatic adenocarcinoma	LPN-01	68	F	na	Left pancreatoctomy (LP)	na[3]	nd[4]	nd	nd	nd	nd
	LPN-03	79	F	na	LP	na	nd	nd	nd	nd	nd
	LPN-08	83	F	na	Cephalo duodenopancreatectomy (CDP)	na	nd	nd	nd	nd	nd
	LPN-11	81	M	na	LP	na	nd	nd	nd	nd	nd
	LPN-14	70	F	na	CDP	na	nd	nd	nd	nd	nd
	LPN-15	53	F	na	CDP	na	nd	nd	nd	nd	nd
	LPN-17	61	M	na	LP	na	nd	nd	nd	nd	nd
	LPN-19	48	M	na	CDP	na	nd	nd	nd	nd	nd
	LPN-21	66	M	na	Total pancreatectomy (TP)	na	nd	nd	nd	nd	nd
	LPN-27	43	F	na	TP	na	nd	nd	nd	nd	nd
	LPN-31	70	F	na	LP	na	nd	nd	nd	nd	nd

1. au, Arbitrary units according to the Diabetes Antibody Standardization Program (Schlosser et al., 2011).
2. Either HLA DR3-DQ2, HLA DR4-DQ8, or both.
3. na, not applicable
4. nd, not determined.
